# Nanofiber membranes for enhanced performance and optimization of proton exchange membrane fuel cells

**DOI:** 10.1126/sciadv.adw5747

**Published:** 2025-09-19

**Authors:** Heng Zhai, Jianuo Chen, Chen Meng, Xiaochen Yang, Zeyu Zhou, Ling Ai, Jiashen Li, Thomas S. Miller, Maria Perez-Page, Stuart M. Holmes

**Affiliations:** ^1^ Department of Chemical Engineering, The University of Manchester, Manchester M13 9PL, UK.; ^2^ Electrochemical Innovation Lab, Department of Chemical Engineering, University College London, London WC1E 7JE, UK.; ^3^ Department of Materials, The University of Manchester, Manchester M13 9PL, UK.

## Abstract

Proton exchange membranes (PEMs) are critical to fuel cell performance, where ion transport, catalyst activity, and mass transfer determine efficiency and durability. However, conventional membranes in PEM fuel cells often suffer from hydrogen crossover, limited conductivity, and poor interfacial stability. To address these challenges, this study develops a nanofiber membrane system with synergistic structural and interfacial enhancement. Through nanofiber architecture and surface engineering, the membrane balances proton conductivity, mechanical strength and electrochemical performance. The sandwich-structure nanofiber membrane (SSNFM) achieves a peak power density of 942 mW cm^−2^ after 100-hour accelerated stress testing, substantially outperforming conventional commercial membranes (520 mW cm^−2^). Electrochemical characterization confirms enhanced proton conductivity for SSNFM (40.4 mS cm^−1^) compared to commercial membranes (17.5 mS cm^−1^). Multiscale analyses, including x-ray computed tomography and multiphase simulations, reveal improved membrane properties, catalyst layer stability, and triple-phase boundary formation, facilitating efficient charge and mass transport. This work presents a membrane design strategy to enhance fuel cell performance in sustainable energy applications.

## Teaser

Porous nanofiber membranes boost proton conductivity, suppress hydrogen crossover, and enhance durability in HT-PEMFCs.

## INTRODUCTION

Proton exchange membrane fuel cells (PEMFCs) have garnered widespread attention as a sustainable and efficient energy technology (*1*–*3*). The membrane electrode assembly (MEA), as the core component of PEMFCs, governs the overall performance of the fuel cell due to the complex multiphase physical and chemical interactions occurring within it (*4*). Optimizing MEAs requires not only improving individual components but also understanding their synergistic interactions within the system. Among these components, proton exchange membranes (PEMs) play a pivotal role in proton conduction, gas separation, and overall fuel cell efficiency. However, the development of innovative fabrication methods for PEMs has lagged behind advances in membrane and catalyst materials (*5*–*8*). Currently, membrane fabrication still predominantly relies on the solution casting method (*9*). While this method offers simplicity and cost-effectiveness, it presents several drawbacks, including difficulties in achieving uniform thickness and challenges in controlling the microstructure, which directly impact the membrane’s performance (*10*). Various polymer modification techniques, such as cross-linking or grafting PEM with other polymers, have been explored to enhance membrane properties. However, these modifications have yet to demonstrate consistent performance and long-term stability to a practical extent (*11*–*13*).

Operating at a higher temperature in the range of 120° to 200°C, high-temperature PEMFCs (HT-PEMFCs) offer distinct advantages over low-temperature PEMFCs (LT-PEMFCs), including the elimination of complex water management systems, tolerance to impure hydrogen fuel sources, and integration of waste heat into combined heat and power systems (*14*, *15*). These features simplify system design and enhance overall efficiency, making HT-PEMFCs ideal for stationary and portable energy applications. In addition, HT-PEMFCs use phosphoric acid (PA)–doped PEMs, where PA acts as the primary proton conductor, replacing the water-dependent conduction mechanism observed in LT-PEMFCs (*16*). Unlike water, which can cause flooding and performance degradation in LT-PEMFCs, PA remains within the membrane at high temperatures, ensuring stable proton transport. However, PA leaching over extended operation remains a critical issue, as it can lead to catalyst layer (CL) degradation and a decline in fuel cell efficiency. The presence of phosphorus (P) in PA provides a unique advantage for characterizing its distribution and migration using x-ray–based techniques, which are not feasible for water in LT-PEMFCs (*17*). These attributes make HT-PEMFCs an optimal platform for studying key fuel cell mechanisms, including triple-phase boundary (TPB) formation, ion transport dynamics, and MEA degradation processes (*18*–*20*).

Given the limitations of current PEM fabrication techniques, electrospinning has emerged as a promising alternative. This versatile technique enables the production of fine nanofibrous matrices with high porosity and uniform web structures, making it widely applicable in fields such as gas separation and water desalination (*21*–*23*). Electrospun nanofiber membranes (NFMs) have also demonstrated considerable potential for application in PEMFCs (*24*, *25*). These membranes offer numerous advantages, including their high porosity, which enhances proton transport, and their large specific surface area, which facilitates effective TPB formation (*26*–*28*). However, their practical implementation remains insufficiently validated, and their exact contributions to PEMFC performance require further investigation beyond simple improvements in TPB development and proton conductivity.

Polybenzimidazole (PBI) membranes, as a representative material for HT-PEMFCs, play critical roles in the MEA including proton conduction, hydrogen crossover prevention, PA retention, and mechanical support (*29*, *30*). The interaction between PBI at the catalyst interface and PA represents a complex system that can be studied using advanced x-ray characterization techniques (*31*, *32*). Integrating electrospun nanofiber–based PBI membranes into HT-PEMFCs presents an opportunity to explore membrane fabrication methods and their effects on fuel cell performance (*33*). Among various x-ray characterization techniques applied to HT-PEMFCs, x-ray computed tomography (CT) enables the three-dimensional reconstruction of MEA structures and provides a powerful tool for diagnosing the effects of different materials on MEA structural changes (*34*). The relatively high atomic number of phosphorus allows x-ray CT to effectively detect PA distribution within the MEA, although segmentation challenges exist due to its grayscale similarity with other components (*35*). Nevertheless, PA leaching can be inferred by analyzing changes in MEA porosity through advanced segmentation techniques.

Here, we fabricated a pure PBI nanofiber mat using electrospinning, achieving a fine fibrous structure with enhanced porosity and proton transport potential. To overcome challenges such as excessive hydrogen permeability and PA flooding, we introduced a surface modification process that optimizes pore sizes and enhances mechanical stability. Furthermore, we developed an innovative sandwich-structure NFM (SSNFM), combining a solution-cast PBI membrane (SCM) with surface-modified nanofiber layers on both sides. This design effectively mitigates hydrogen crossover while preserving the high PA storage capacity and improving the TPB for electrochemical reactions. The x-ray CT and multiphase/multiphysics visualization simulations revealed the morphological stability of the SSNFM and demonstrated its ability to maintain a well-defined CL structure under operating conditions, substantially reducing PA leaching and CL degradation compared to commercial PBI membranes. Simulations also provided detailed insights into gas diffusion, water distribution, and electrochemical reaction zones, highlighting the improved oxygen transport and enhanced proton pathways within the SSNFM. By validating the exceptional durability of the SSNFM under a simulated heavy-duty vehicle accelerated stress testing (AST) protocol, this study not only establishes its potential for transport applications but also addresses critical issues (e.g., PA retention, hydrogen crossover, and long-term stability) that are equally important for stationary and portable HT-PEMFC systems. This work represents a breakthrough in overcoming the limitations of PBI-based membranes and offers a scalable design strategy for next-generation high-performance fuel cell technologies.

## RESULTS

### Surface modification of pristine NFM

**Fig. 1.**
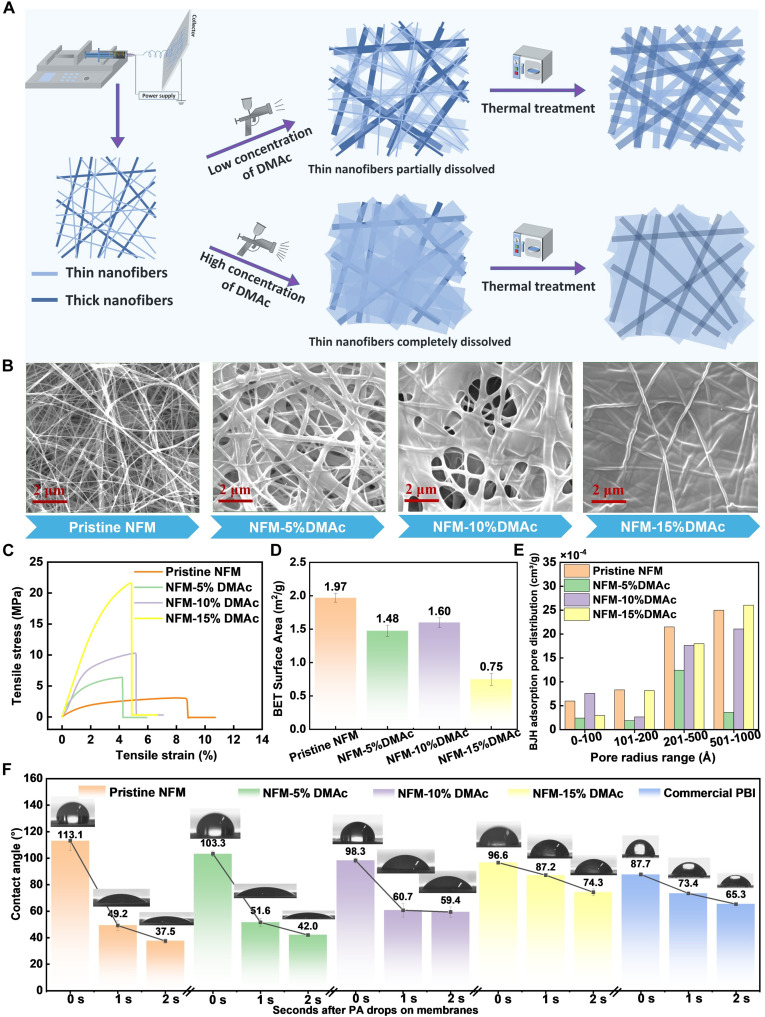
Surface-modified porous PBI NFM. (**A**) Schematic illustration shows fabrications of pristine NFM and surface-modified NFMs. (**B**) Scanning electron microscopy (SEM) images exhibit surface morphologies of pristine NFM and surface-modified NFMs. (**C**) Mechanical properties of various NFMs. (**D**) BET-specific surface area of various NFMs. (**E**) BJH adsorption pore distributions of various NFMs. (**F**) PA contact angle test of NFMs and commercial PBI membrane, showing remarkable PA adsorbing capability of NFMs.

The thickness of nanofibers in PBI NFM mats produced by electrospinning ranged from several nanometers to hundreds of nanometers as shown in fig. S1. The thin nanofibers were dissolved when a low-concentration dimethylacetamide (DMAc) solution was sprayed on the NFM surface and subsequently cross-connected after thermal treatment in a vacuum oven as illustrated in Fig. 1A. This dissolving-and-casting process generated a fibrous network with a complex mesh architecture on the NFM. More thin nanofibers dissolved as a higher-concentration DMAc solution was applied, leading to smaller pore sizes on NFM surface. The thermogravimetric analysis (TGA) indicated that this modification slightly reduces the thermal stability of the membranes due to the plasticizing effect of DMAc, which increases polymer chain mobility (fig. S2). However, since HT-PEMFCs operate at temperature ranging from 120° to 200°C, well below the degradation onset (⁓465°C), this has no notable impact on practical performance. Figure 1B compared surface morphologies of NFM treated with various DMAc concentrations (digital images of various NFM membranes can be found in fig. S3). Thin nanofibers were completely dissolved with no obvious pores observed on the NFM treated with 15% DMAc solvent (NFM-15%DMAc). This interconnected structure substantially enhanced the mechanical strength of the surface-modified NFM. As shown in Fig. 1C, the tensile strength of NFM-15% DMAc has increased by a factor of 10 compared to pristine NFM. The Brunauer-Emmett-Teller (BET) surface area analysis in Fig. 1D revealed that the NFM-10%DMAc (NFM treated with 10% DMAc) exhibited the highest specific surface area among the surface-modified NFMs due to the presence of more numerous small micron pores distributed on membrane surface (fig. S4). Furthermore, the Barrett-Joyner-Halenda (BJH) adsorption pore distribution in Fig. 1E indicated a higher proportion of micropores smaller than 100 Å on NFM-10%DMAc.

PA, as an excellent proton conductor, plays a crucial role in facilitating the transport of protons from the anode to the cathode through the membrane (*36*). Consequently, the amount of PA doped into the NFM membrane directly influences proton conductivity in a fuel cell. Table S1 summarizes the volume and area swelling properties, as well as acid doping level (ADL) of various NFMs (calculation methods of PA uptake rate and ADL can be seen in eq. S1 and S2). It revealed that the pristine NFM has the highest ADL (54.41 mol) due to the extensive space within its porous structure. However, this open structure compromises its mechanical stability compared to commercial PBI membranes when doped with excessive acid, resulting in severe PA loss during fuel cell operation. In contrast, surface-modified NFMs, such as NFM-5%DMAc (ADL 27.14 mol) and NFM-10%DMAc (ADL 15.30 mol), demonstrate incredible PA doping capabilities, far exceeding that of the commercial PBI membrane (ADL 9.23 mol). This is further supported by the PA contact angle test shown in Fig. 1F, where the contact angles of NFM-5%DMAc and NFM-10%DMAc rapidly decrease within 2 s after acid is applied to the membrane surface, highlighting their superior PA absorption capacity. Meanwhile, Fourier transform infrared spectroscopy (FT-IR) in fig. S5 also reveals a broader carbonyl stretch with a shift to higher wave number in surface-modified NFM membranes, indicating increased hydrogen bonding, which contributes to enhanced PA retention. This improvement in proton transport arises not only from higher acid uptake but also from strengthened acid retention mechanisms introduced by surface structural modification at the molecular level. Specifically, DMAc-induced surface densification reduces free volume and enhances hydrogen bonding between the polymer and PA. The compact surface architecture restricts acid diffusion pathways, while increased polar interactions stabilize the doped acid under operating conditions. Together, these structural and chemical modifications provide a clear molecular basis for the improved PA retention, durability, and proton conductivity observed in NFM-10%DMAc.

### Electrochemical evaluation of surface-modified NFM MEAs

**Fig. 2.**
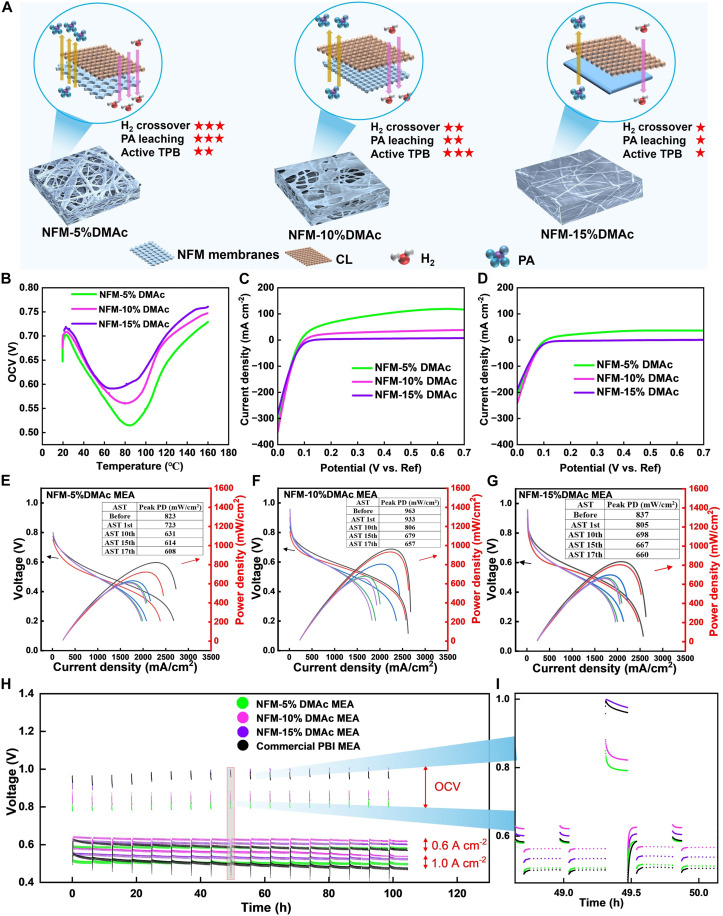
Electrochemical evaluation of surface-modified NFM MEAs, 160°C, anode: H_2_ (100 ml min^−1^), and cathode: O_2_ (100 ml min^−1^). (**A**) Schematic illustration of surface morphology of NFM membranes, showing their H_2_ crossover and PA leaching properties. (**B**) OCV changes of fuel cell heating to 160°C. (**C**) LSV before AST. (**D**) LSV after 100-hour AST. (**E** to **G**) Polarization curves during AST, (E) NFM-5%DMAc, (F) NFM-10%DMAc, and (G) NFM-15%DMAc. (**H** and **I**) A continuous 100-hour AST plot and voltage details at the 50th hour.

Hydrogen crossover is the undesired diffusion of hydrogen through the fuel cell membrane from anode to cathode (*37*). Similarly, PA leaching refers to the loss of PA from the membrane, leading to decreased proton conductivity of the membrane and reduced active catalytic sites (*38*). These challenges limit the applications of NFMs in HT-PEMFCs due to their inherently open and porous surface structure. However, modifying the surface morphology of pristine NFM can mitigate these issues. As illustrated in Fig. 2A, hydrogen crossover and PA migration in the CL and NFM membranes are influenced by the pore size. Narrower pores within the NFM membrane reduce opportunities for hydrogen crossover and PA leaching. For instance, NFM-15%DMAc, with its smooth, pore-free surface, effectively blocks hydrogen permeation and PA loss. However, this flat surface design drawbacks, including a reduced specific surface area and fewer contact sites between the membrane and catalysts, limiting the formation of effective TPB. Conversely, the complex mesh texture on the surface of NFM-10%DMAc offers a higher specific surface area, promoting the generation of more active size of TPB. Figure 2B compares the open-circuit voltage (OCV) of NFM MEAs during heating the fuel cell fixture to 160°C. The lower OCV in NFM-5%DMAc compared to other NFM MEAs indicates more hydrogen crossover and reduced fuel utilization, thereby reducing the voltage output. The linear sweep voltammetry (LSV) plots before AST in Fig. 2C further confirm these findings, showing hydrogen crossover rate of 1.43 × 10^−6^, 5.35 × 10^−7^, and 5.96 × 10^−8^ mol s^−1^ for NFM-5%DMAc, NFM-10%DMAc, and NFM-15%DMAc, respectively (table S2, eq. S3, and the Supplementary Materials). By generating more complex mesh texture on the NFM surface, hydrogen crossover can be substantially reduced. After 100 hours of AST, these values further decreased to 5.17 × 10^−7^, 3.34 × 10^−8^, and 2.69 × 10^−8^ mol s^−1^, respectively (Fig. 2D). This improvement in hydrogen crossover resistance can be attributed to consistent mechanical pressure in the high-temperature fuel cell system during AST, leading to the compression and thinning of the NFM structure, as confirmed by the following CT scanning results. On the other hand, membrane compression may also introduce potential drawbacks. First, a reduction in membrane thickness could limit the overall PA storage capacity, potentially accelerating PA loss under prolonged operation. This concern becomes more pronounced if compression continues beyond the tested AST window, as the membrane’s volume available for PA retention would diminish despite its high PA affinity (*14*). Second, while moderate compression may enhance the formation of effective TPBs, excessive densification could reduce the fibrous membrane’s surface roughness and interfacial complexity. As a result, the NFM may gradually lose its distinct nanofibrous characteristics and behave more like a conventional solution-cast membrane, ultimately compromising long-term electrochemical performance.

Figure 2 (E to G) displays the polarization curves of NFM-5%DMAc, NFM-10%DMAc, and NFM-15%DMAc MEAs across 100-hour ASTs, respectively. All the NFM MEAs exhibit higher peak power density both before and after AST compared to the commercial PBI MEAs (fig. S6). This improvement is attributed to the higher ADL and enhanced TPB interactions. Among the treated membranes, the NFM-10%DMAc MEA showed the highest power density of 963 mW cm^−2^ before AST (Fig. 2C), representing a 34% increase compared to the commercial PBI MEA (717 mW cm^−2^). Additional PA-humidity coupled condition test on the NFM-10%DMAc further revealed that prolonged PA doping reduced tensile stress, likely due to increased acid uptake compromising the membrane’s mechanical integrity. Conversely, exposure to water vapor (i.e., simulated humidity) resulted in increased tensile stress, suggesting partial PA migration out of the membrane, which reduced plasticization and slightly restored stiffness (fig. S7). The AST was designed to simulate the harsh conditions that accelerate the ageing process to evaluate the durability and performance stability of fuel cells. During the AST, the applied potential cycling induced PA migration from regions of high to low concentration, which reduced the peak power density of NFM-10%DMAc to 657 mW cm^−2^ after 100 hours. Despite this decline, the post-AST peak power density remained considerably higher than that of the commercial PBI MEA (520 mW cm^−2^). The NFM-15%DMAc MEA, while showing a lower initial peak power density of 837 mW cm^−2^, demonstrated superior resistance to performance deterioration during AST due to its ability to mitigate PA leaching. In particular, from the 10th cycle to the end of the AST (roughly 40 hours onward), its performance degradation was minimal, with a decrease of only 38 mW cm^−2^ (Fig. 2D). Figure 2H illustrates the continuous 100-hour AST curves, highlighting performance variations of the NFM MEAs and the commercial PBI MEA at OCV and current densities of 0.6 A and 1.0 A cm^−2^. The NFM MEAs consistently exhibited enhanced performance and long-term durability compared to the commercial PBI MEA, as further detailed in the 50-hour AST plots in Fig. 2I. In summary, the NFM-15%DMAc MEA maintained the highest OCV, while the NFM-10%DMAc MEA demonstrated superior voltage outputs at current density of 0.6 A and 1.0 A cm^−2^ throughout the AST. The detailed trend of voltage deterioration presented in fig. S8 and corresponding calculated voltage degradation rates in table S3 confirmed the exceptional stability and durability of NFM MEAs, with the voltage degradation rates of 0.68, 0.94, and 1.78% at current density of 0.6 A cm^−2^ for NFM-5%DMAc, NFM-10%DMAc, and NFM-15%DMAc MEA, respectively, all substantially lower than commercial PBI MEA’s rate of 7.42%.

Electrochemical impedance spectroscopy (EIS) reveals key electrochemical characteristics of the fuel cell, including proton conductivity, charge transfer resistance, and mass transport behavior, offering critical insights for optimizing performance and durability (*39*). The Nyquist plots from EIS illustrated in fig. S9A depict the electrochemical characteristics of commercial PBI MEA and NFM MEAs before and after 100-hour AST, with equivalent circuit model used for fitting shown in fig. S9B. For all NFM MEAs, the Nyquist plots display two distinct semicircles, corresponding to charge transfer resistance and mass transfer resistance of the anode (high-frequency region) and cathode (low-frequency region). In contrast, the commercial PBI MEA shows a single large semicircle, indicating less differentiation between these processes. This difference can be attributed to the unique 3D porous structure and fibrous morphology of the NFM, which creates well-defined and spatially separated interfaces between the membrane and the CLs on both sides. The improved interfacial distinctness allows the anode and cathode responses to be more easily resolved electrochemically. Conversely, the commercial PBI membrane likely forms a more homogeneous and interpenetrated interface with the CLs, leading to an overlap of impedance features in the frequency domain and resulting in a single broad semicircle. It is important to note that, under our EIS conditions (galvanostatic EIS at a constant DC current of 3 A and sinusoidal AC current of 0.1 A), mass transfer resistance can only be accurately recorded in the very low-frequency range (typically below 1 Hz) (*40*, *41*). As a result, distinguishing between charge transfer resistance and mass transfer resistance in this setup is challenging. The initial ohmic resistance (*R*_ohm_) of NFM MEAs (85 milliohms for NFM-5%DMAc, 64 milliohms for NFM-10%DMAc, and 81 milliohms for NFM-15%DMAc) is notably lower than that of the commercial PBI MEA (120 milliohms), indicating better proton conductivity. After AST, the sum of charge transfer and mass transfer resistance [*R*_Δ(*c+m*)_] in commercial PBI MEA has increased substantially from 118 milliohms to 170 milliohms, reflecting severe deterioration of CL, likely due to PA leaching and suboptimal interlayer contact. These issues have been mitigated in NFM MEAs, as evidenced by the minimal increase in *R*_Δ(*c+m*)_. Among the NFM MEAs, NFM-10%DMAc exhibits the lowest *R*_ohm_ and *R*_Δ(*c+m*)_ both before and after AST, confirming its superior initial performance and long-term stability under AST conditions.

### The morphology and pore analysis of surface-modified NFM MEAs

**Fig. 3.**
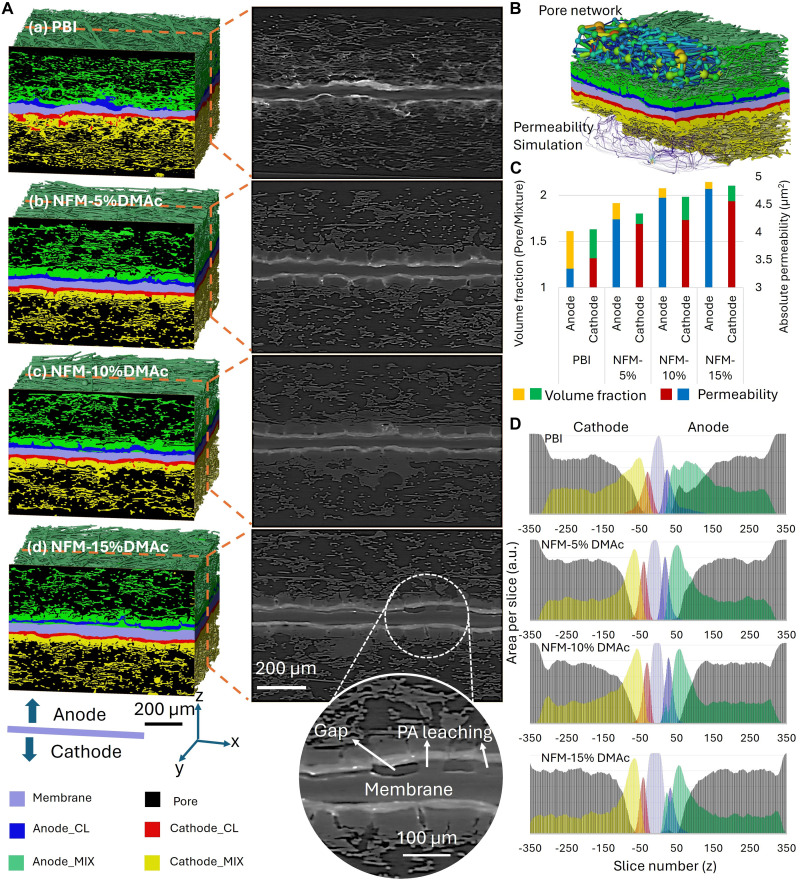
The components and their distribution in MEAs based on different membranes after AST. (**A**) 3D segmentation and 2D grayscale images of MEAs. (**B**) Illustration of pore network and permeability simulations. (**C**) Average pore fraction and absolute permeability of MEAs. (**D**) Slice-by-slice components area fraction distribution.

To investigate the impact of different membrane structures on the morphology of MEAs and the subsequent component migration, x-ray CT was used. Figure 3 illustrates representative 3D structures of MEAs based on the different membranes after 100-hour AST. Through segmentation, the 3D structures of the membranes, CLs, and pores were extracted. The remaining components were collectively defined as a mixed phase, which includes constituents such as carbon fibers, carbon black, binders, and PA. Because of the higher atomic number of phosphorus in PA compared to the elements commonly found in water, PA is detectable in x-ray CT imaging. While it is not possible to fully isolate PA individually based on grayscale intensity, the extent of PA leaching can be inferred through variations in the mixed phase and pore distribution (*4*). As shown in Fig. 3A, MEAs based on different membranes exhibit distinct characteristics. MEAs constructed with commercial PBI membranes demonstrate pronounced CL migration, resulting in a disordered structure. In contrast, MEAs with NFM membranes maintain a well-defined layered structure of the CL with relatively uniform thickness. This observation suggests that NFM membranes effectively buffer the CL against structural disruptions caused by mechanical pressure, gas flow, and PA leaching, thereby preserving the overall morphology of the MEA. NFMs treated with varying concentrations of DMAc exhibit notable differences in performance. MEAs based on NFM-5%DMAc membranes demonstrate excellent adhesion between the CL and the membrane. However, as the DMAc concentration in the surface treatment increases, the adhesion between the CL and the membrane slightly diminishes. This highlights the buffering role of NFM, which facilitates effective contact between the membrane and the CL, contributing to the structural stability and performance of the MEA. Although Fig. 3A (a to d) demonstrates that increasing the DMAc content during the surface treatment of NFM increases the likelihood of membrane-CL delamination, a closer examination of the grayscale magnification in Fig. 3A (d) reveals some notable findings. Specifically, while the cross-section of the MEA based on the NFM-15%DMAc membrane exhibits localized separation between the membrane and the CL, the unique properties of NFM-15%DMAc mitigate the effects of this separation. The membrane’s finely tuned PA affinity, coupled with the capillary action at the membrane-catalyst and membrane surface interfaces, enables the gap between the membrane and the CL to be bridged by PA connections. This PA bridging phenomenon, observed as a grayscale connection between the membrane and CL in Fig. 3A (d), indicates a retained ionic pathway despite local delamination. While this partially separated interface may raise concerns about mechanical stability, the presence of PA within the gap likely maintains proton conductivity and mitigates performance loss (*4*). However, such a structure may also present risks under extended cycling, such as PA redistribution or evaporation, potentially affecting long-term durability. Notably, this PA bridging behavior is absent in MEAs based on conventional PBI membranes, underscoring the distinctive interfacial characteristics of the NFM-15%DMAc system.

The pore network and absolute permeability simulations based on CT images shown in Fig. 3B provide insights into the porosity and permeability of MEAs based on different membranes. As illustrated in Fig. 3C, the permeability and porosity of MEAs based on NFM are markedly higher than those of MEAs using commercial PBI membranes, with permeability showing a positive correlation with porosity to some extent. Moreover, with more DMAc participating in the NFM surface modification, both porosity and permeability gradually improve. These changes in porosity and permeability are probably related to the migration of components such as PA. This hypothesis can be further validated by analyzing the slice-by-slice area fraction of components. Figure 3D demonstrates that the mixed phase in MEAs based on NFM membranes is more concentrated toward the membrane center compared to those based on commercial PBI membranes, with pronounced differences around slices ±50. In addition, a comparison of NFM-based MEAs reveals that, within the regions between ±100 and ±250 slices for the anode and cathode, the mixed-phase content decreases progressively as the DMAc content increases from 5 to 15%. This change in the mixed phase is primarily attributed to the migration of PA and the accompanying redistribution of other components. Although the negative impact of component migration on the permeability of the gas diffusion media in the NFM-15%DMAc-based MEA is relatively minor, the increased tendency for delamination between the membrane and the CL in this system, as mentioned earlier, is noteworthy. This is corroborated by the mixed-phase distribution of the NFM-15%DMAc system in Fig. 3D, where a small peak in the mixed phase is observed near slice 25. This indicates that certain regions between the membrane and the CL are filled with the mixed phase. These findings suggest that variations in DMAc content during treatment have different impacts on gas diffusion and membrane-catalyst interactions, necessitating a balanced optimization of both factors. This dual effect also explains the differences in performance and EIS results observed among MEAs based on various NFM membranes.

### Fabrication of SSNFM MEA and performance improvement

**Fig. 4.**
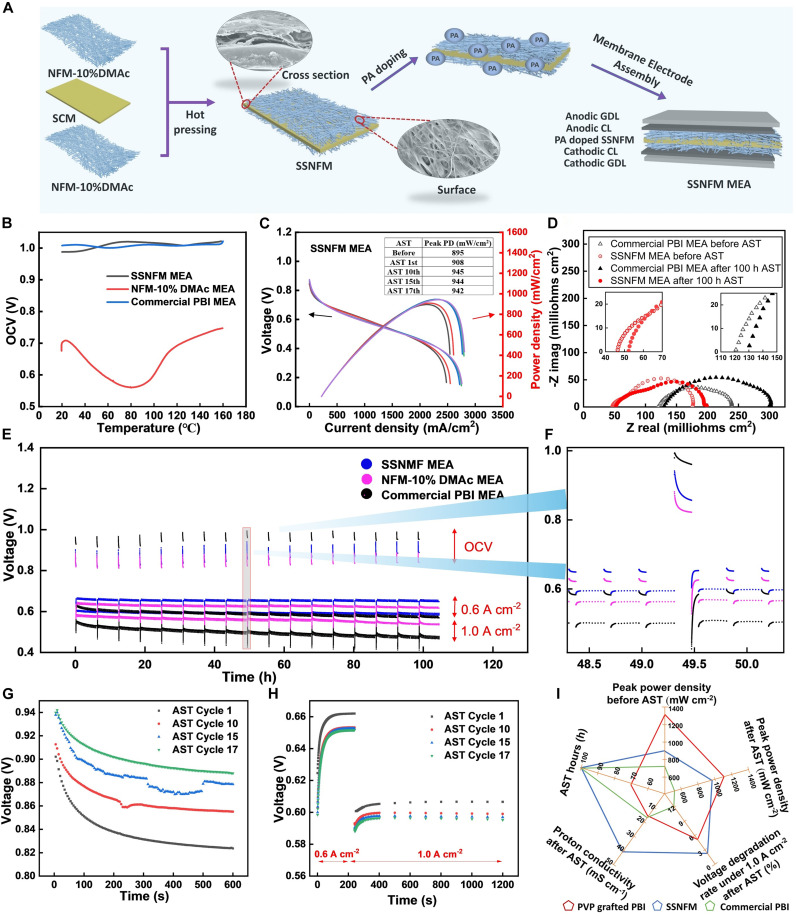
Electrochemical evaluations of SSNFM MEA, 160°C, anode: H_2_ (100 ml min^−1^), and cathode: O_2_ (100 ml min^−1^). (**A**) Schematic illustration of fabrication of SSNFM MEA. (**B**) OCV changes of fuel cell heating to 160°C. (**C**) Polarization curves of SSNFM MEA during 100-hour AST (equals to 17 cycles). (**D**) Nyquist plots of SSNFM and commercial PBI MEA before and after AST. (**E** and **F**) A continuous 100-hour AST and voltage profiles around the 50th hour. (**G**) OCV changes of SSNFM MEA during AST. (**H**) Voltages under current densities of 0.6 A cm^−2^ and 1.0 A cm^−2^ during AST. (**I**) Performance comparison among commercial PBI, SSNFM, and PVP grafted PBI MEA (calculation method of proton conductivity can be seen in eq. S4).

The superior performance of NFM-10%DMAc establishes it as a promising PEM for HT-PEMFCs. However, its porous structure still poses a challenge by allowing hydrogen crossover, limiting further performance enhancements. To address this issue, an SSNFM has been developed, as depicted in Fig. 4A. The SSNFM consists of two layers of NFM-10%DMAc flanking a SCM in the center. The three membranes were hot-pressed at high temperature and pressure to form the innovative sandwich structure. The dense SCM layer serves as a hydrogen crossover barrier, while the NFM-10%DMAc layers provide abundant active sites for electrochemical reactions. After doping in PA, the electrochemical properties of the SSNFM MEA were evaluated using a Gamry potentiostat with a booster. As shown in Fig. 4B, the initial OCV of the SSNFM (⁓1.0 V) is notably higher than that of NFM-10%DMAc (⁓0.7 V) and is comparable to the commercial PBI MEA, demonstrating a substantial reduction in hydrogen crossover. This improvement was further validated by LSV results in fig. S10. The polarization curves in Fig. 4C illustrate an enhanced peak power density both before and after the AST. The SSNFM MEA exhibits a performance increase after 100-hour AST, with peak power density improving from 895 to 942 mW cm^−2^. This counterintuitive phenomenon suggests a possible in operando activation effect. We hypothesize that the elevated operating conditions during AST induce mild compression and densification of the SSNFM, leading to enhanced membrane-electrode interface contact and reduced interfacial resistance. In addition, the redistribution and stabilization of PA may promote the formation of more continuous proton-conducting networks, particularly within the finely structured CL-membrane interface (*17*). The fibrous architecture of the NFM layers further facilitates mechanical adaptability, ensuring better retention of TPB integrity and catalyst accessibility. These combined effects appear to contribute to improved proton transport and oxygen reduction kinetics, thus explaining the observed post-AST performance enhancement. Figure S11 further demonstrates its performance stability under H_2_/Air conditions with the same temperature and gas feeding rate. The SSNFM MEA maintained higher and more stable peak power density (270 mW cm^−2^, 100-hour AST) and voltage output over the full 100-hour AST, whereas the commercial PBI MEA (228 mW cm^−2^, 48-hour AST) showed rapid degradation and failed the full AST before 48 hours. These results validate the SSNFM’s robust oxygen transport capability and its practical potential for heavy-duty vehicle applications.

The Nyquist plots in Fig. 4D show the substantially lower ohmic resistance of the SSNFM MEA both before and after AST, attributed to its ample PA storage capacity and improved CL-membrane contact. The slightly higher initial charge and mass transfer resistance [*R*_Δ(*c+m*)_] of the SSNFM MEA (129 milliohms) compared to that of a commercial PBI MEA (118 milliohms) can be attributed to high-pressure assembly conditions, which expelled excess PA from the membrane into the CL. However, after AST, the *R*_Δ(*c+m*)_ for SSNFM increased modestly to 148 milliohms, whereas it rose to 170 milliohms in the commercial PBI MEA (table S4), demonstrating the SSNFM’s superior ability to mitigate PA leaching. The improved durability is attributed to its notably higher initial PA content (26.95 mg cm^−2^) and better retention (24.99 mg cm^−2^ after AST; retention rate, 92.7%) compared to the commercial PBI MEA (19.11 to 15.15 mg cm^−2^; retention rate, 79.3%) as shown in table S5. The 100-hour AST results in Fig. 4E underscore the remarkable performance stability of the SSNFM MEA. Compared to NFM-10%DMAc MEA, the SSNFM exhibited notably improved OCV and higher voltage outputs at cyclic current densities of 0.6 A cm^−2^ and 1.0 A cm^−2^ (Fig. 4F). Voltage profiles over 17 AST cycles (Fig. 4, G and H) show a steady increase in OCV and minimal voltage degradation at cyclic current densities of 0.6 and 1.0 A cm^−2^. The voltage degradation rate of the SSNFM MEA under 1.0 A cm^−2^ is only 1.81%, a marked improvement compared to the commercial PBI MEA (11.4%) and the NFM-10%DMAc MEA (6.32%), as summarized in table S6. The performance comparison in Fig. 4I and table S7 further substantiates the SSNFM’s outstanding stability and performance, surpassing many PBI-based membranes reported in the literature (*4*, *8*, *12*, *34*, *42*–*44*).

### Multiphase and multiphysics visualization simulations of different MEAs after AST

**Fig. 5.**
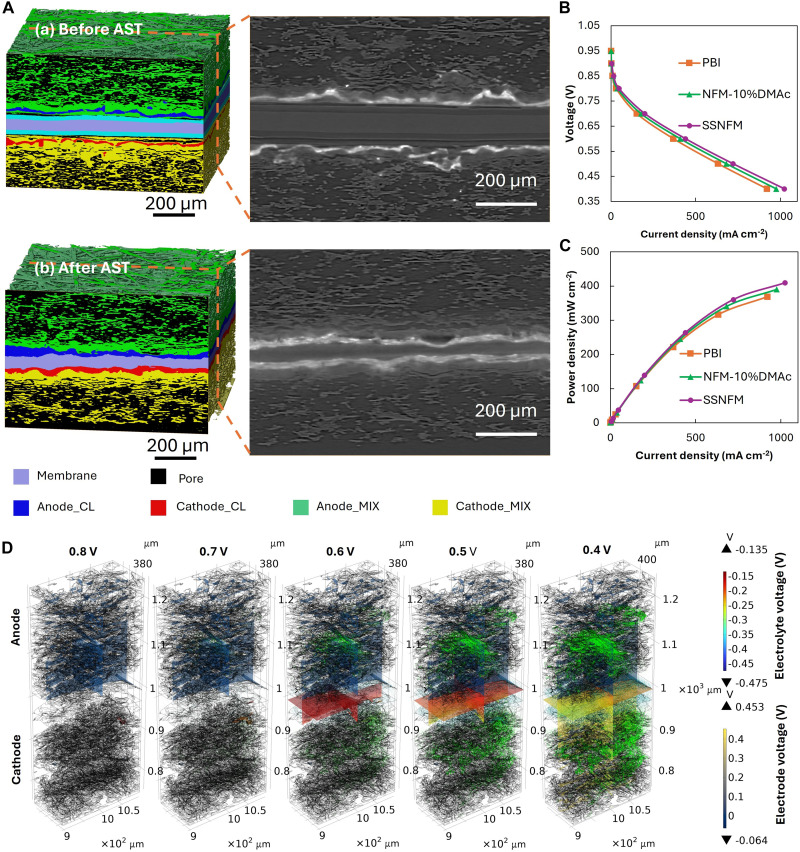
CT images and corresponding multiphase and multiphysics simulations. (**A**) CT images of SSNFM-based MEA before and after AST. (**B**) Simulated polarization curves of different MEAs based on different membranes after AST. (**C**) Simulated power density curves of different MEAs based on different membranes after AST. (**D**) The potential and current density vector distribution of the PBI-based MEA after AST [arrow volume: electrode (green) and electrolyte (cyan) current density vector].

The differences exhibited by the SSNFM compared to the NFM were further investigated through x-ray CT scans of the 3D structure of the MEA based on the SSNFM membrane, as shown in Fig. 5A. Before AST, the fresh MEA based on SSNFM displayed clear stratification between the NFM and SCM layers. Figure 5A demonstrates that NFM and SCM can achieve excellent adhesion. However, after AST, the membrane became thinner under applied pressure, and this stratification feature was no longer evident. After AST, NFM and SCM exhibit improved integration, while the NFM layers on both sides continue to provide effective buffering between the membrane and the CL. This results in excellent adhesion between the membrane and the CL.

Electrochemical testing indicates that the performance differences among MEAs based on commercial PBI membranes, NFM, and SSNFM are largely influenced by hydrogen crossover. To investigate the impact of different membranes on the electrochemical processes of MEAs while excluding the effects of hydrogen crossover, multiphase and multiphysics simulations using COMSOL were used. These simulations generated polarization curves and power density curves, as shown in Fig. 5 (B and C), demonstrating that hydrogen crossover is not the sole factor affecting performance. The simulated electrochemical performance was derived by analyzing the potential and current distribution across the electrolyte and electrode at various voltages, as illustrated in Fig. 5D. In addition, the multiphase and multiphysics framework, which incorporates processes such as gas mole fraction and water distribution, will be detailed in the following sections. As shown in Fig. 5 (B and C), while the simulated electrochemical performance does not exactly match the experimental results, the relative trends are consistent. This indicates that the performance enhancement of SSNFM compared to NFM-10%DMAc in MEAs is not solely attributed to its improvement in mitigating hydrogen crossover but also to its influence on the multiphase and multiphysics processes within the MEA. Furthermore, the simulated performance is slightly lower than the experimentally measured performance. This discrepancy arises because, to more clearly demonstrate the effect of structural variations on oxygen mole fraction within the MEA, air was used as the cathode inlet gas in the simulation instead of pure oxygen. This will be elaborated upon in greater detail in the following sections.

**Fig. 6.**
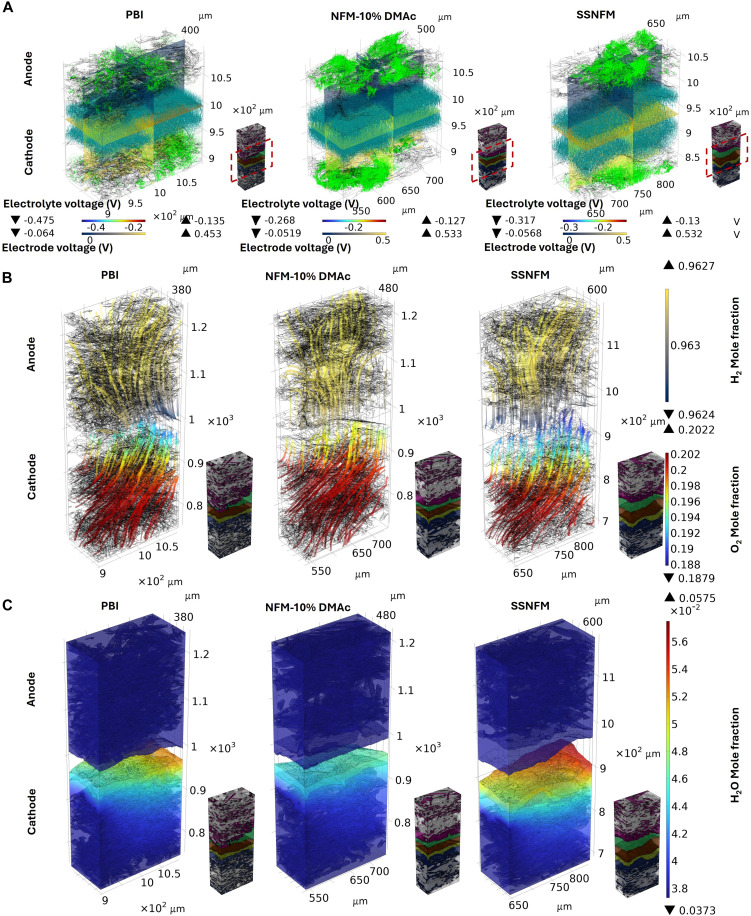
Multiphase and multiphysics visualization simulations of MEAs based on different membranes after AST. (**A**) The potential and current density vector distribution of the different membranes-based MEA [arrow volume: electrode (green) and electrolyte (cyan) current density vector]. (**B**) Gas mole fraction distribution of the different membranes-based MEA. (**C**) Water mole fraction distribution of the different membranes-based MEA.

A comparative visualization of multiphase and multiphysics simulations for MEAs based on different membranes provides deeper insights into the mechanisms driving electrochemical performance differences. The distribution of electrolyte and electrode current vectors, as well as potential near the membrane region, is illustrated in Fig. 6A. For the MEA based on the commercial PBI membrane, the severe degradation of the CL structure after AST results in notably lower electrode current density compared to MEAs based on NFM-10%DMAc and SSNFM. While the NFM-10%DMAc-based MEA exhibits more uniform membrane and catalyst structures and a correspondingly regular distribution of current density across the electrolyte and electrode, the SSNFM-based MEA achieves enhanced performance through a distinct mechanism. In the SSNFM-based MEA, the compression-induced curvature changes at the membrane-catalyst interface not only maintain strong adhesion between the membrane and the catalyst but also expand the TPB of the electrolyte, catalyst, and gas. This expanded TPB creates additional pathways for proton transport, as represented by the current density vectors. This effect is evident in the broader distribution of electrolyte current vectors in the SSNFM-based MEA, highlighting its superior performance potential. Previous permeability simulations have demonstrated that the electrode permeability of the NFM-10%DMAc-based MEA is superior to that of the PBI-based MEA. As a result, the oxygen mole fraction in the CL region of the NFM-10%DMAc-based MEA is higher than that of the PBI-based MEA (Fig. 6B), leading to lower mass transfer resistance. However, despite the improved electrochemical performance, the SSNFM-based MEA also exhibits a lower oxygen mole fraction in the CL region. This observation requires a comprehensive analysis in conjunction with the current vector distribution and water distribution shown in Fig. 6 (A and C). Figure 3A shows that after AST, the CL structure of the PBI-based MEA suffers localized damage. As a result, Fig. 6A reveals a regional concentration of current density vectors in the cathode of the PBI-based MEA, leading to localized water accumulation, which in turn affects the oxygen mole fraction in these regions. In contrast, the SSNFM-based MEA exhibits a more uniform and extensive distribution of current density vectors, indicating higher overall reaction activity. This also results in greater water production, as evidenced by the higher overall water content in the cathode of the SSNFM-based MEA as shown in Fig. 6C. Consequently, the lower oxygen mole fraction in the cathode of the SSNFM-based MEA is a combined result of higher oxygen consumption and increased water content generated by the reaction. This behavior highlights the optimization of the TPB within the catalyst region of the SSNFM-based MEA, contributing to its superior performance.

## DISCUSSION

This study presents a comprehensive approach to addressing key limitations in HT-PEMFCs through the development of an advanced membrane system. By integrating surface-modified nanofiber layers with a dense solution-cast PBI core, the membrane achieves a balance between proton conductivity, hydrogen crossover resistance, and mechanical stability. This design leverages high PA retention and enhanced TPB properties of the nanofiber layers while ensuring structural robustness under operating conditions.

Electrochemical evaluations demonstrate the superior performance of this sandwich-structure membrane system, with a peak power density of 942 mW cm^−2^ after 100 hours of AST, substantially surpassing commercial PBI membranes. Multiscale characterizations, including x-ray CT and multiphase simulations, reveal improvements in interfacial contact, reduced component migration, and enhanced electrochemical reaction zones. These coordinated enhancements underscore the importance of a system-level design approach that optimizes the interactions between membrane, CL, and reactant transport.

The findings provide valuable insights into the mechanisms underlying membrane-catalyst interactions and establish a foundation for designing high-performance HT-PEMFCs. By addressing both material and interfacial challenges, this work offers a scalable and robust solution for advancing clean energy technologies, contributing to the development of more efficient and durable fuel cell systems. These advances align with the broader goals of sustainable energy transition and support the practical deployment of next-generation energy conversion technologies.

## MATERIALS AND METHODS

### Materials

PBI/DMAc solution provided by PBI Performance Products Company was diluted into appropriate concentrations for the fabrication of PBI NFMs and solution casting membranes (SCM). To compare the performance differences, we used commercial PBI membranes (PBI Performance Products Inc., USA). The microporous layer (MPL) and CL were fabricated by spraying MPL ink (Ketjen EC-300J) and Pt/C catalyst ink (60% Pt on high surface area advanced carbon support, Fuel Cell Store) on a carbon paper (THPH 090, Toray) in sequential order. The 150-μm-thick polytetrafluoroethylene (PTFE) gasket and PA (85%) were obtained from Goodfellow Cambridge Ltd. and Thermo Fisher Scientific, respectively.

### Electrospinning of pristine NFM

The electrospinning solution was prepared by dissolving 10 weight % (wt %) PBI and 1 wt % LiCl in 89 wt % DMAc by stirring at 70 rpm for 24 hours at ambient conditions. Ten-milliliter electrospinning solution was loaded into a syringe equipped with a 21-gauge needle and injected at a constant rate of 0.5 ml/hour. A roll collector spinning at 200 r/min was used to collect electrospun nanofibers. The distance between the nozzle tip and collector remained at 25 cm, with an applied voltage at 23 kV. Throughout the electrospinning process, the environmental conditions inside the chamber were strictly controlled at 40°C with a relative humidity of 30%. Last, a NFM mat with an average thickness of 80 ± 10 μm was achieved.

### Surface modification of NFM

The porous NFM mat was post-treated by spraying a total amount of 2 ml of ethanol/DMAc solution on both sides of a 4 cm by 4 cm NFM. After spraying, the membranes were placed in an oven at 60°C for 10 min under vacuum. The ratio of ethanol and DMAc are 95:5, 90:10, and 85:15 to for the preparation of NFM-5%DMAc, NFM-10%DMAc, and NFM-15%DMAc, respectively.

### Fabrication of SCM

Six-milliliter 2% (wt %) PBI/DMAc solution was poured into a glass dish with diameter of 9 cm. The solution was placed in an oven at 80°C for 24 hours and then at 130°C for 3 hours under vacuum to remove the DMAc solvent. In the end, a thin membrane with thickness of 15 ± 3 μm was prepared.

### Fabrication of SSNFM

The SCM was clamped by two NFM-10%DMAc membranes on each side. The whole sandwich-like membrane was hot-pressed at temperature of 140°C and pressure of 80 psi for 4 min to assemble a SSNFM.

### Preparation of MEA

The MPL with loading of Ketjen Black (1 mg/cm^2^) and CL with loading of Pt catalysts (1 mg/cm^2^) were spray-coated on the carbon paper sequentially, forming electrodes with effective area of 5 cm^2^. The membranes were soaked in 85% PA at room temperature for 20 hours to achieve PA-doped membranes. The electrodes and membranes covered by PTFE films (supported as gaskets) were hot-pressed at temperature of 140°C and pressure of 80 psi for 4 min to assemble the MEA.

### Characterizations of membranes and electrochemical characterizations of MEAs

The BET surface area was analyzed on an ASAP2020 using N_2_ adsorption. The surface morphology was characterized by Quanta250 scanning electron microscope (SEM). The TGA of membranes was performed using the thermal analyzer (Q50, TA Instruments, USA). Membranes were heated in the range of 25° to 800°C at a heating rate of 10°C min^−1^ in a nitrogen atmosphere at flow rate of 50 ml min^−1^. FT-IR was conducted with a Nicolet iS 5 FT-IR spectrometers (Thermo Fisher Scientific). The electrochemical performance of MEA was evaluated using a fuel cell fixture (Scribner, effective area of 5 cm^2^) connected to an electrochemical workstation (Gamry, Reference 3000 with 30K booster). The measurements were performed at an operating temperature of 160°C. For H_2_/O_2_ testing, hydrogen and oxygen gases were supplied at flow rates of 100 ml min^−1^ each. For the H_2_/air testing conditions, hydrogen and air were similarly introduced at flow rates of 100 ml min^−1^. The polarization curves were obtained by discharging from OCV to 0.1 V in current increments of 0.05 A. The Nyquist plots were acquired by running the Galvanostatic EIS at a constant DC current of 3 A and sinusoidal AC current of 0.1 A, spanning the frequency range from 10 kHz to 0.1 Hz. To evaluate membrane durability under realistic load conditions, we adopted an AST protocol involving repeated cycles of 0.6 A cm^−2^ (4 min) and 1.0 A cm^−2^ (16 min) current operation, followed by a 10-min OCV rest, completing one AST cycle (6.17 hours) out of the full 17 AST cycles (approximately 105 hours). According to our previous studies, the voltage degradation induced by this AST protocol was found to be approximately 24 times greater than that observed under the corresponding constant current density (*45*). This method is consistent with recently proposed fuel cell durability testing strategies that simulate field-relevant dynamic loads in heavy-duty vehicle applications (*46*). The LSV measurement used a linear potential sweep scanning from 0 to 0.7 V by feeding nitrogen gas at cathode and hydrogen gas at anode of the fuel cell.

### PA-humidity coupled conditioning test

NFM-10%DMAc membranes were first doped in PA at 160°C for 24 and 48 hours to simulate in operando chemical exposure. Subsequently, the membranes were exposed to water vapor for 5, 10, and 20 min by suspending them over boiling water (fig. S12), mimicking humidity-induced swelling and acid redistribution effects. The treated membranes were then subjected to tensile testing using a universal testing machine (Instron 3344) to assess changes in mechanical strength under these coupled chemical-mechanical stress conditions (fig. S7).

### MEA titration

The PA retention in MEA was evaluated by acid-base titration (*47*). The commercial PBI MEA and SSNFM MEA, before or after 100-hour ASTs were dried at 60°C for 12 hours and subsequently immersed in deionized water for 15 hours under ambient conditions. This solution was titrated with 0.05 M NaOH using methyl orange as an indicator. The calculation method is provided in eq. S5.

### 3D scanning, reconstruction, and visualization simulations of MEAs

Circular discs (2 mm diameter) were extracted from the active area using a laser micromachining system (Oxford Lasers, A Series/Compact System) at 0.6 W power and 0.5 mm/s scan speed, with five passes. Micro-CT imaging was performed using a Zeiss Xradia 620 Versa, achieving 1 μm voxel resolution with a 4× objective at 60 kV tube voltage and 8 s exposure time. The CT data were cropped (1016 pixels by 1016 pixels by 508 pixels) in Avizo, filtered for clarity, and segmented using artificial intelligence algorithms to isolate the membrane, CL, pores, and a mixed phase (carbon particles, fibers, PTFE binder, and PA). Porosity and connectivity analyses were conducted, and a reduced volume (100 pixels by 100 pixels by 50 pixels) was prepared for COMSOL multiphysics simulations to optimize computational efficiency.

Simulation in COMSOL focuses on analyzing mass and momentum transport of reactants and electrochemical currents under steady-state conditions for different MEA structures. The model incorporates hydrogen fuel cell interfaces, free and porous media flow, and multiphysics coupling of gas-phase reactions and flow dynamics. Key electrochemical reactions are described using the Butler-Volmer equation for the hydrogen oxidation and oxygen reduction reactions, with additional mass and momentum transport modeled via the Maxwell-Stefan equation, Navier-Stokes equations, and Darcy’s Law. Specific boundary conditions are tailored to the unique 3D structures of the MEAs. The key parameters used in the simulation are presented in table S8. For detailed methodology, refer to our previously published work (*34*).
