# Supplementary material for: Nanofiber membranes for enhanced performance and optimization of proton exchange membrane fuel cells
Source: Sci Adv. 2025 Sep 19;11(38):eadw5747. doi: 10.1126/sciadv.adw5747 (PMC12448063; doi:10.1126/sciadv.adw5747)
Supplement: Supplementary file 1 — Supplementary Text Figs. S1 to S12 Tables S1 to S8 [file sciadv.adw5747_sm.pdf]

Science Advances
AAAS

Supplementary Materials for

Nanofiber membranes for enhanced performance and optimization of proton exchange membrane fuel cells

Heng Zhai *et al.*

Corresponding author: Stuart M. Holmes, stuart.holmes@manchester.ac.uk; Jianuo Chen, jianuo.chen@ucl.ac.uk

Sci. Adv. **11**, eadw5747 (2025)
DOI: 10.1126/sciadv.adw5747

This PDF file includes:

Supplementary Text
Figs. S1 to S12
Tables S1 to S8

Supplementary Text

The calculation of PA uptake rate and ADL follows the equation below,

$$PA_{uptake} = \frac{W_{wet}}{W_{dry}} \times 100\% \tag{S1}$$

$$ADL = \frac{(W_{wet} - W_{dry})/M_{PA}}{W_{dry}/M_{PBI}} \tag{S2}$$

Where W_{wet} and W_{dry} are the membrane weight after and before acid doping at room temperature, respectively. M_{PA}, M_{PBI} are the mole weight of phosphoric acid, and polybenzimidazole repeat unit respectively.

The calculation of hydrogen crossover follows the equation below,

$$n_{H_2Crossover} = \frac{j_{H_2crossover} \times A}{n \times F} \tag{S3}$$

Where A is the fuel cell active area, n is the number of electrons taking part in the reaction (2 e^- per H_2 molecule), and F is Faraday's constant (96,485 C/mol).

The calculation of proton conductivity follows the equation below,

$$\sigma = \frac{L}{R \times A} \tag{S4}$$

Where σ is the proton conductivity (S/cm), L is the membrane thickness (cm), R is the membrane internal resistance (Ω), and A is the membrane active area (cm^2). The membrane internal resistance was obtained from the test result of electrochemical impedance spectroscopy (EIS).

The calculation of PA retention in MEA follows the equation below,

$$PA\ retention\ (mg/cm^2) = \frac{V_{NaOH} \times C_{NaOH} \times M_{W,PA}}{Equiv_{mol} \times A} \tag{S5}$$

Where V_{NaOH} is the volume of NaOH, C_{NaOH} (mol L^{-1}) is the molar concentration of NaOH used, $M_{w,PA}$ is the molecular weight of PA (98 g mol^{-1}), $Equiv_{mol}$ is the equivalent mole of titrant for PA (1 in this case for methyl orange indicator), and A (cm^2) is the actual area of the immersed sample.

Supplementary Figures

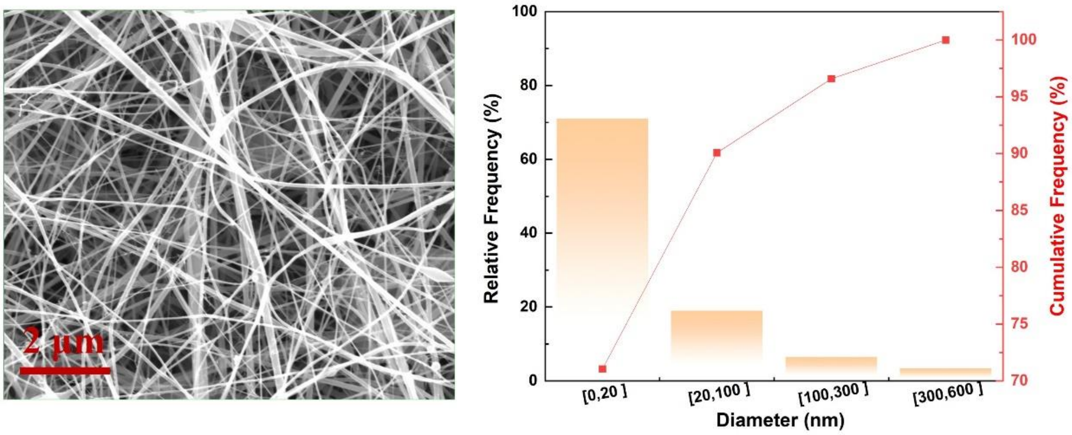

Fig. S1. SEM images of NFM and nanofiber diameter distributions.

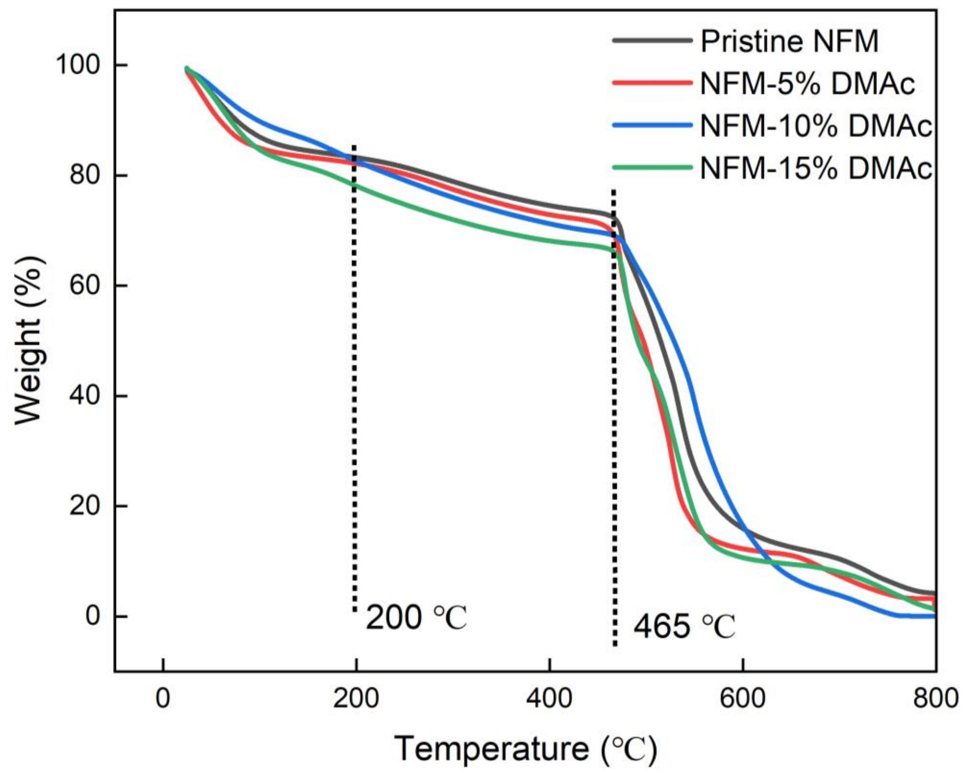

Fig. S2. TGA analysis of NFM membranes. The TGA results show that the weight loss of the pristine NFM membrane occurs at approximately 465 °C, corresponding to the decomposition of benzimidazole groups. In contrast, surface-modified NFM membranes exhibit weight loss at lower temperatures due to the plasticizing effect of DMAc, which increases polymer chain mobility and reduces thermal stability. However, it is important to note that the typical operating temperature range of HT-PEMFCs (120–200 °C) falls well below the decomposition onset, meaning there is no significant difference in thermal performance between pristine and surface-modified NFM membranes under actual working conditions.

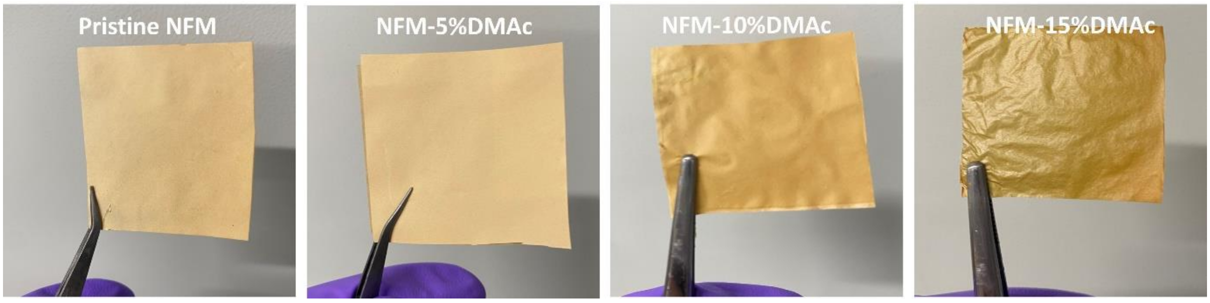

Fig. S3. Digital images of pristine NFM and surface-modified NFMs.

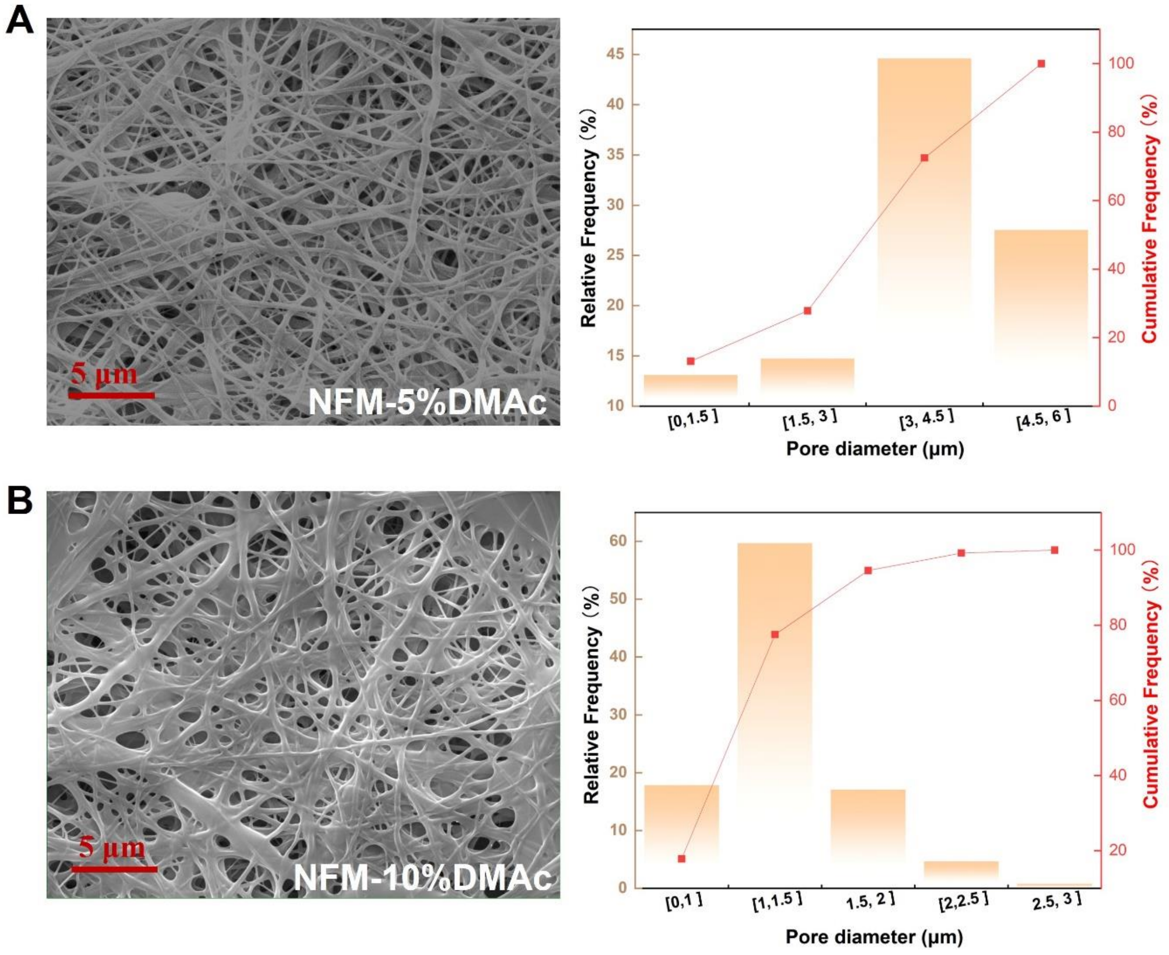

Fig. S4. Pore diameter distributions of NFM membranes.

A. NFM-5%DMAc.

B. NFM-10%DMAc.

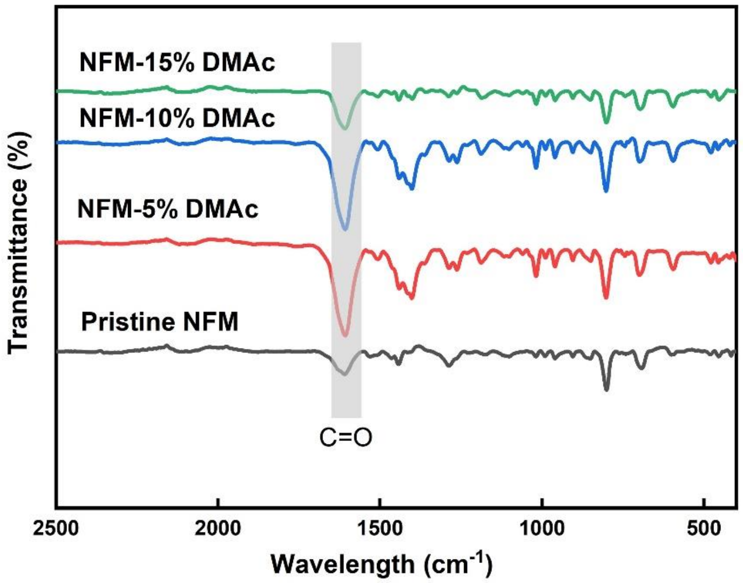

Fig. S5. FTIR analysis of NFM membranes. The carbonyl (C=O) stretch at 1650 cm^{-1} in surface-modified NFM membranes broadens and shifts to higher wavenumber, indicating increased hydrogen bonding, which enhances PA adsorption capacity.

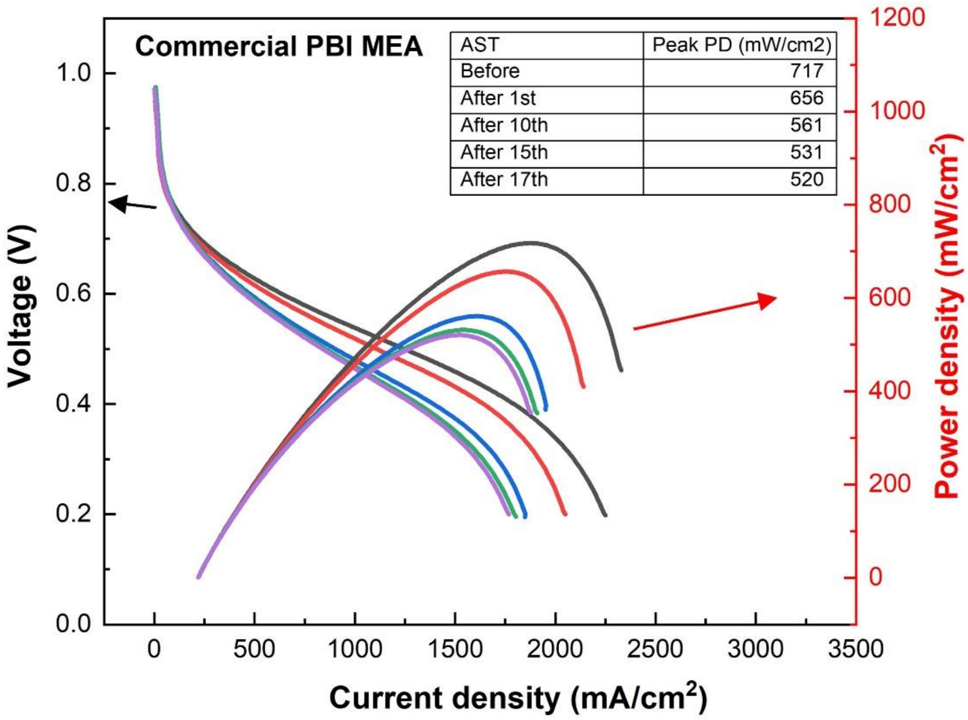

Fig. S6. Polarization curves of commercial PBI MEA before and after AST.

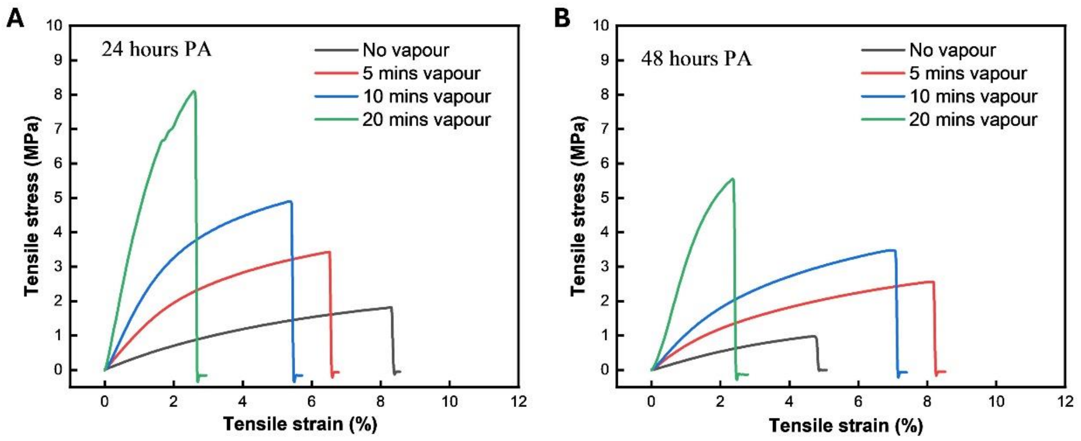

Fig. S7. Mechanical properties of the NFM-10%DMAc membrane after varying PA doping durations and subsequent humidity treatments.

A. PA doping duration of 24 hours and subsequent humidity treatments of 0, 5, 10, and 20 minutes.

B. PA doping duration of 48 hours and subsequent humidity treatments of 0, 5, 10, and 20 minutes.

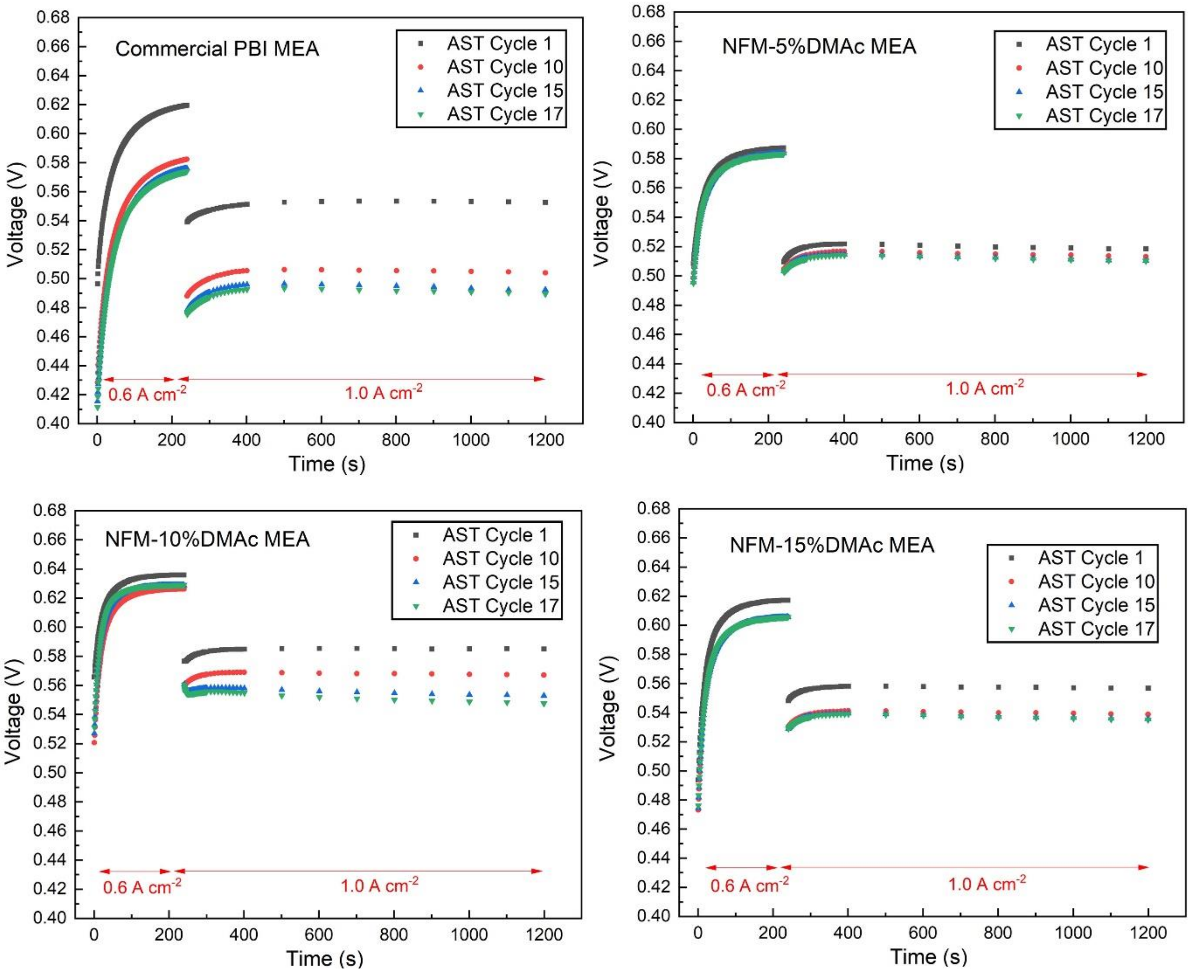

Fig. S8. The voltages under current densities of 0.6 A cm^{-2} and 1.0 A cm^{-2} during 100 h AST of commercial PBI MEA and NFM MEAs.

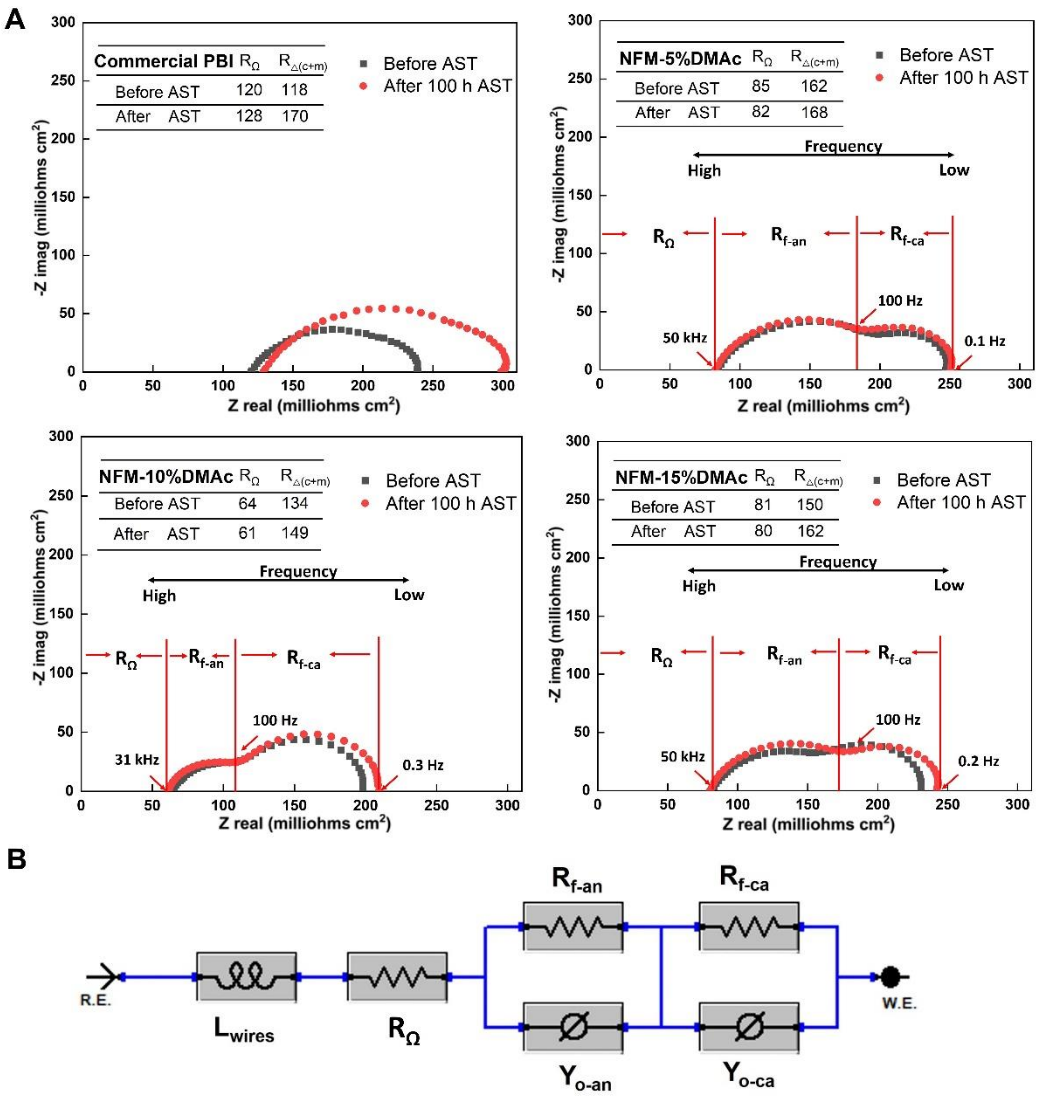

L_{wires} (H): Inductance of cables

R_{Ω} (ohm): Ohmic resistance

$R_{f\text{-}an}$ (ohm): Charge transfer resistance and mass transfer resistance of the anode

$Y_{o\text{-}an}$ (S s^{a}): Constant phase element of the anode

$R_{f\text{-}ca}$ (ohm): Charge transfer resistance and mass transfer resistance of the cathode

$Y_{o\text{-}ca}$ (S s^{a}): Constant phase element of the cathode

a: Dimensionless exponent

Fig. S9. The Nyquist curves of commercial PBI MEA and NFM MEA and their equivalent circuits.

A. The Nyquist curves. The frequency data inside the plots are all corresponding to the Nyquist plots after AST.

B. The equivalent circuit and definition of parameters.

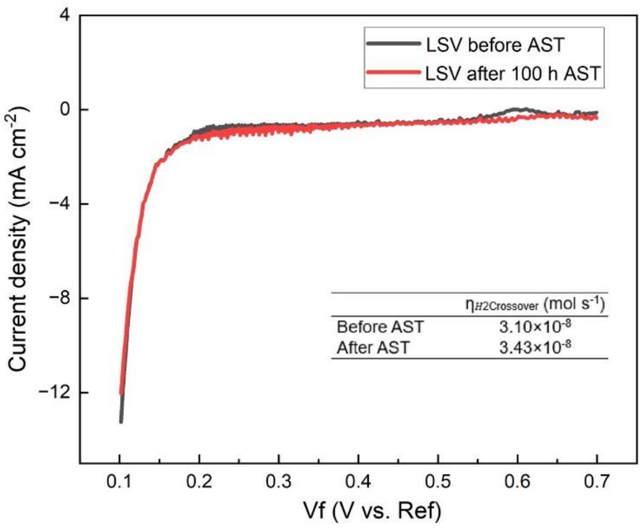

Fig. S10. LSV curves of a SSNFM MEA before and after AST.

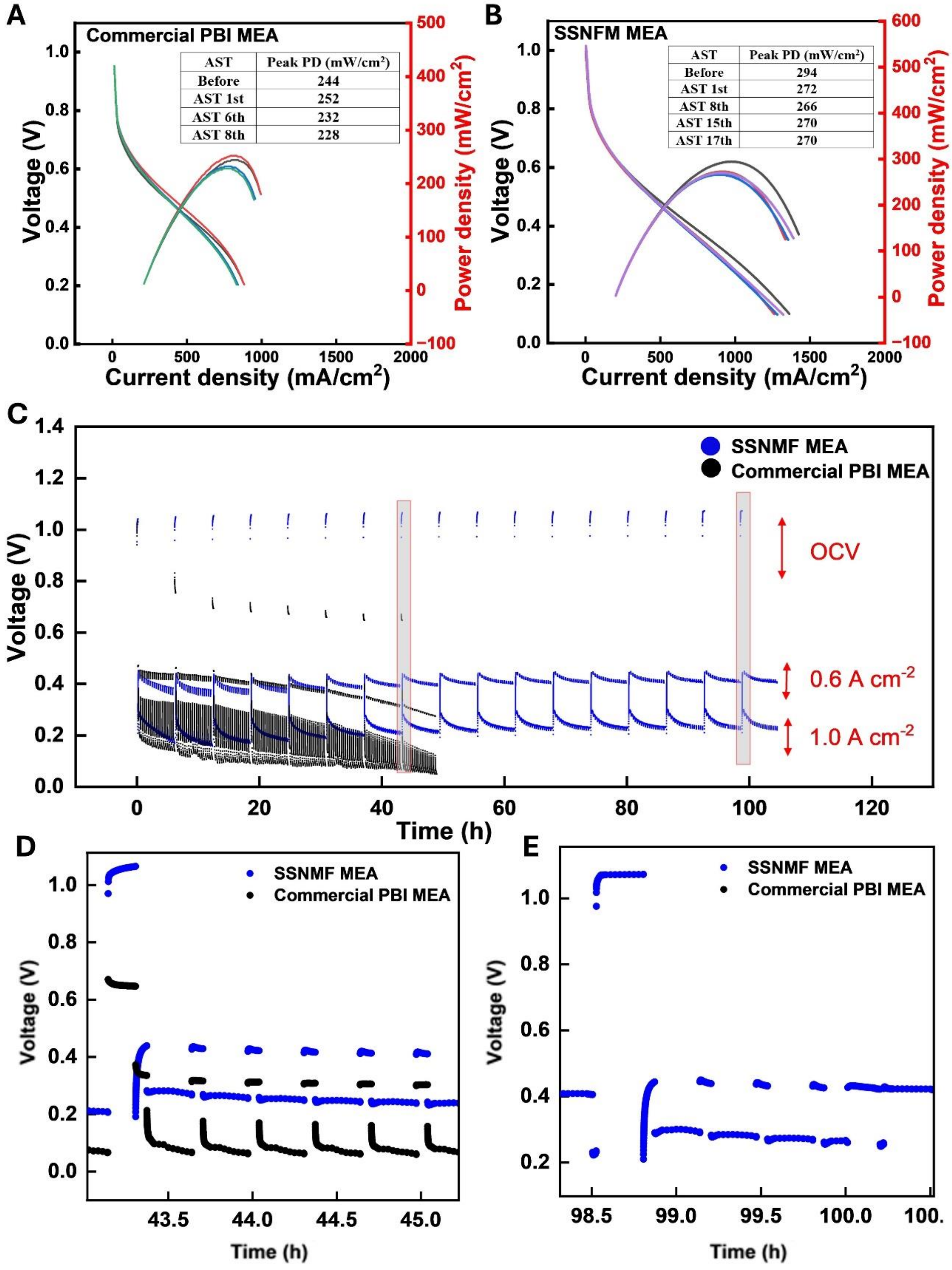

Fig. S11. Electrochemical evaluation of SSNFM and commercial PBI MEA under H_2/Air conditions, 160 °C, anode: H_2 (100 mL min^{-1}), cathode: Air (100 mL min^{-1}).

A. Polarization curve of commercial PBI MEA during 48-hour AST (the rapid performance degradation prevented completion of the planned 100-hour AST).

B. Polarization curve of the SSNFM MEA during 100-hour AST.

C. Performance of commercial PBI MEA and the SSNFM MEA after AST.

D. Detailed voltage profiles around the 44th hour.

E. Detailed voltage profiles around the 99th hour.

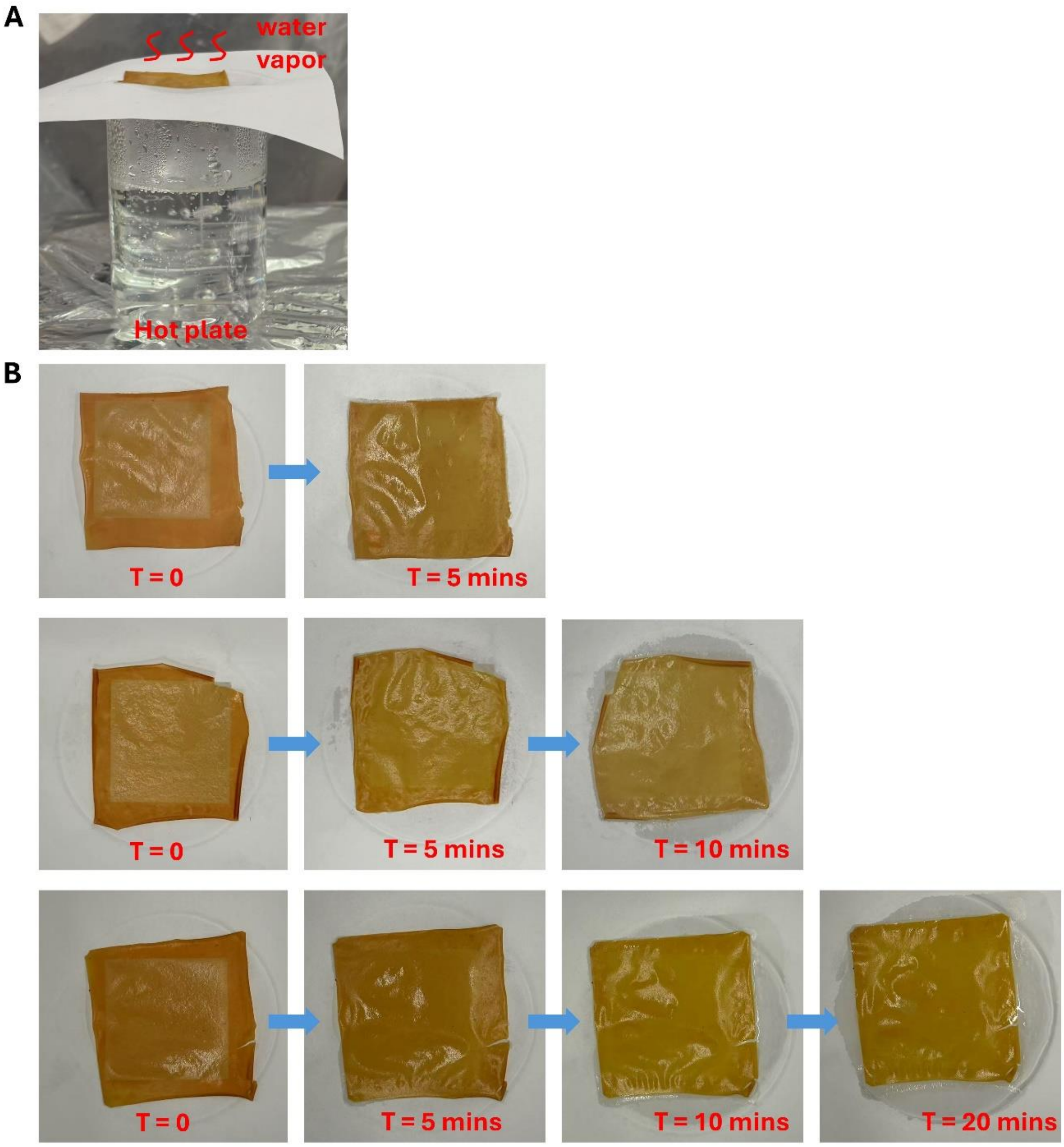

Fig. S12. Durability of the NFM-10%DMAc membrane subjected to different PA doping durations and humidity treatments.

A. Digital images of experimental setup of the NFM-10%DMAc membrane treated by humidity.

B. Digital images of the NFM-10%DMAc membrane doped in PA for 24 hours, followed by humidity treatments of 5, 10, and 20 minutes.

Supplementary Tables

Table S1

Comparisons of different PBI membranes in acid doping properties

Membrane	Thickness before acid doping (μm)	Thickness after acid doping (μm)	Acid uptake (%)	Volume swelling (%)	Area swelling (%)	ADL (mol)
NFM	110±5	N/A	1730.9%	N/A	N/A	54.41
NFM-5%DMAc	100±5	190±5	863.7%	80.7%	5.5%	27.14
NFM-10%DMAc	90±5	130±5	486.7%	82.0%	23.5%	15.30
NFM-15%DMAc	85±5	120±5	265.6%	45.2%	22.8%	8.35
Commercial PBI	50±5	80±5	293.8%	152.6%	46.8%	9.23

Table S2

The calculated hydrogen crossover

MEAs	$\eta_{H2\mathrm{Crossover}}$ before AST (mol s^{-1})	$\eta_{H2\mathrm{Crossover}}$ after AST (mol s^{-1})
NFM-5%DMAc	1.43×10^{-6}	5.17×10^{-7}
NFM-10%DMAc	5.35×10^{-7}	3.34×10^{-8}
NFM-15%DMAc	5.96×10^{-8}	2.69×10^{-8}

Table S3

The calculated voltage degradation rates

MEAs	Voltages at current density of 0.6 A cm^{-2} (mV)			Voltages at current density of 1.0 A cm^{-2} (mV)		
	Before AST	After AST	Degradation rate (%)	Before AST	After AST	Degradation rate (%)
Commercial PBI MEA	620	574	7.42%	553	490	11.40%
NFM-5%DMAc	587	583	0.68%	518	510	1.54%
NFM-10%DMAc	636	630	0.94%	585	548	6.32%
NFM-15%DMAc	617	606	1.78%	557	535	3.95%

Table S4

The ohmic, mass and charge resistance before and after AST

MEAs	Before AST		After AST	
	R_{ohm}	$R_{\triangle(c+m)}$	R_{ohm}	$R_{\triangle(c+m)}$
Commercial PBI MEA	120	118	128	170
SSNFM MEA	46	129	52	148

Table S5

PA retention in MEA before and after AST

PA retention in MEA	Commercial PBI MEA	SSNFM MEA
Before AST (mg/cm^2)	19.11	26.95
After 100-hour AST (mg/cm^2)	15.15	24.99
PA retention rate (%)	79.3%	92.7%

Table S6

The calculated voltage degradation rates

MEAs	Voltages at current density of 0.6 A cm^{-2} (mV)			Voltages at current density of 1.0 A cm^{-2} (mV)		
	Before AST	After AST	Degradation rate (%)	Before AST	After AST	Degradation rate (%)
Commercial PBI MEA	620	574	7.42%	553	490	11.40%
NFM-10%DMAc	636	630	0.94%	585	548	6.32%
SSNFM MEA	662	652	1.51%	606	595	1.81%

Table S7

Performance comparison of PBI-based HT-PEMFCs

Membrane	Pt loading (mW cm^{-2})	Feed gas (mL min^{-1})	PPD (mW cm^{-2})	PPD after test (mW cm^{-2})	Durability test: Voltage degradation rate (mV h^{-1})	Durability test: Test Condition	Test duration (h)
NFM-5%DMAc **(This work)**	1.0	H_2/O_2, 100/100 0 % RH	823	608	0.0818 at 1.0 A cm^{-2}	AST process: 4 min at 0.6 A cm^{-2} and 16 min 1.0 A cm^{-2}, then 10 min OCV, 160 ºC	100
NFM-10%DMAc **(This work)**			963	657	0.3736 at 1.0 A cm^{-2}		
NFM-15%DMAc **(This work)**			837	660	0.2164 at 1.0 A cm^{-2}		
SSNFM **(This work)**			895	942	0.1132 at 1.0 A cm^{-2}		
Commercial PBI			717	520	0.5240 at 1.0 A cm^{-2}		
PBI-g-PVP 30 *(12)*	1.0	H_2/O_2, 200/200, 0 % RH	1312	1105	0.476 at 1.0 A cm^{-2}	AST process: 4 min at 0.6 A cm^{-2} and 16 min 1.0 A cm^{-2}, then 10 min OCV, 160 ºC	70
porous OPBI *(42)*	0.65	H_2/O_2, 200/200, 0 % RH	485.3	N/A	5.42	constant current density ,0.3 A cm^{-2}, 160 °C	150
p-OPBI-ATMP (*8*)	1.2 ± 0.1	H_2/O_2, 150/200, 0 % RH	980	N/A	0.00546	constant current density of 0.2 A cm^{-2}, 160 ºC	180
PBI-SLG (*4*)	1.0	H_2/O_2, 100/100 0 % RH	321	480	1.71 at 1.0 A cm^{-2}	AST process: 4 min at 0.6 A cm^{-2} and 16 min 1.0 A cm^{-2}, then 5 min OCV, 150 ºC	60
40%-OPBI *(43)*	0.6	H_2/O_2, 80/160 0 % RH	1090.5	N/A	0.0132	constant current density of 0.2 A cm^{-2}, 160 ºC	200
Three-layer-OPBI *(44)*	1.0	H_2/O_2, 80/160 0 % RH	604.6	N/A	0.73	constant current density of	

Laser scribed PBI *(34)*	1.0	anode: H_2 ($\lambda = 1.2$), cathode: O_2 ($\lambda = 2.0$), 60°C	734.3	817.2	N/A	0.2 A cm^{-2}, 160 ºC AST process: 4 min at 0.6 A cm^{-2} and 16 min 1.0 A cm^{-2}, then 10 min OCV, 160 ºC	37

Table S8

Key parameters for simulation

Name (unit)	Value			Description
	PBI	NFM-10% DMAC	SSNFM	
T (°C)		160		Cell temperature
σ_{Ω} $(S\ m^{-1})$	6.25	20.3	19.2	Membrane conductivity (from EIS)
$\varepsilon_{gas,gdl}$	Anode:1.61<br>Cathode:1.63	Anode:2.07<br>Cathode:1.98	Anode:2.13<br>Cathode:2.09	Gas diffusion layer gas pore volume fraction
$\varepsilon_{gas,mix}$	$=\varepsilon_{gas,gdl}$			Mixture phase gas pore volume fraction
$\varepsilon_{gas,cl}$	$=\varepsilon_{gas,cl}$			Catalyst layer gas pore volume fraction
$\kappa_{p,gdl}$ (μm^2)	Anode:3.34<br>Cathode:3.53	Anode:4.62<br>Cathode:4.22	Anode:4.73<br>Cathode:4.64	Gas diffusion layer permeability
$\kappa_{p,mix}$ (μm^2)	$\kappa_{p,gdl}/6.25$			Mixture phase permeability
$\kappa_{p,cl}$ (μm^2)	$\kappa_{p,gdl}/6.25$			Catalyst layer permeability
